# Mild disease course of SARS-CoV-2 infections and mild side effects of vaccination in Pompe disease: a cohort description

**DOI:** 10.1186/s13023-022-02268-y

**Published:** 2022-03-04

**Authors:** G. Ismailova, M. J. Mackenbach, J. M. P. van den Hout, A. T. van der Ploeg, E. Brusse, M. A. E. M. Wagenmakers

**Affiliations:** 1 Center for Lysosomal and Metabolic Diseases, Department of Pediatrics, Erasmus University Medical Center, Rotterdam, The Netherlands; 2 Center for Lysosomal and Metabolic Diseases, Department of Neurology, Erasmus University Medical Center, Rotterdam, The Netherlands; 3 Center for Lysosomal and Metabolic Diseases, Department of Internal Medicine, Erasmus University Medical Center, Rotterdam, The Netherlands

**Keywords:** Pompe disease, Glycogen storage disease type II, SARS-CoV-2, COVID-19, COVID-19 vaccines

## Abstract

**Introduction:**

Patients with Glycogen Storage Disease type II (GSDII), an inheritable metabolic myopathy also known as Pompe disease, are considered to be at risk for severe COVID-19 due to a reduced respiratory function and a tendency to be overweight. However, so far little is known about the course of SARS-CoV-2 infection and side effects of COVID-19 vaccinations in patients with GSDII.

**Methods:**

169 Dutch Pompe patients are followed at the Erasmus MC Rotterdam. During the COVID-19 pandemic patients were requested to directly inform their physicians about SARS-CoV-2 infection. Infected patients were interviewed regularly by telephone until their symptoms subsided. Furthermore, all patients eligible for vaccination on 16-7-2021 (≥ 17 years, n = 122) were asked to complete a questionnaire.

**Results:**

To date, fifteen patients (8.9% of our cohort) reported a SARS-CoV-2 infection (classic infantile Pompe disease n = 5, late onset n = 10). No patients were admitted to hospital or needed intensivation of ventilatory support. All patients made a recovery within 19 days. 41.8% of patients filled in our questionnaire regarding vaccination, of whom 98% were vaccinated. Besides one case of perimyocarditis, only mild side effects were reported.

**Conclusion:**

Overall, patients with Pompe disease showed mild symptoms from infection with SARS-CoV-2. All patients made a full recovery. Side effects after vaccination were mostly mild.

## Introduction

As of 1st of November 2021, a total of 248 million cases with SARS-CoV-2 have been confirmed [1]. Risk factors for a severe course of COVID-19, the disease caused by SARS-CoV-2 infection, include older age, male gender, respiratory disease and obesity [2–4]. In general patients with neuromuscular disease are potentially vulnerable to respiratory infections due to frequent cardiorespiratory involvement and comorbidities [5]. Pompe disease or Glycogen Storage Disease type II (GSDII) is a rare neuromuscular disease. It is a metabolic myopathy, caused by a reduced activity of the enzyme alpha-glucosidase, which leads to progressive skeletal muscle weakness. It affects the respiratory muscles and leads to mobility problems, increasing the likelihood of obesity. The clinical spectrum of patients with Pompe disease is broad. Patients with the classic infantile subtype are severely affected with hypotonia and hypertrophic cardiomyopathy from the first months of life, whereas patients with late onset Pompe disease (LOPD) manifest a slowly progressive course with onset of symptoms between early childhood and the last decades of life [6]. Enzyme replacement therapy (ERT) has improved the prospects but most patients exhibit residual disease [7]. As stated by the World Muscle Society Group, patients with Pompe disease are considered to be at medium to high risk of severe COVID-19, due to impaired mobility, sarcopenia and lower respiratory function [8–11]. Furthermore, patients with classic infantile Pompe disease sometimes receive immune modulating therapy to prevent the formation of antibodies against ERT via B-cell depletion which may lead to persistent and severe COVID-19 [11–13].

In this paper, we will share the outcomes of SARS-CoV-2 infections as well as data on side effects of COVID-19 vaccination in patients with Pompe disease.

## Methods

The Center for Lysosomal and Metabolic Diseases (CLMD) of Erasmus University Medical Center, Rotterdam, is the national reference center for patients with Pompe disease in the Netherlands. We treat 169 patients with Pompe disease (classic infantile form n = 21, LOPD n = 146) of whom 125 are treated with ERT. Thirty-two percent of patients are either in part or fully dependent on ventilators due to a reduced respiratory function (“Appendix”). During the pandemic, three classic infantile Pompe patients were immune compromised, with three receiving intravenous immunoglobulins (IVIG) and one being treated with Rituximab (“Appendix”).

From the beginning of the pandemic, all patients were explicitly asked to report a SARS-CoV-2 infection to their treating physician in order to monitor them with regular interviews by telephone and, in case they receive home infusion therapy with ERT, make adjustments when needed. The aim of the interviews was to gain insight in the course of infection, such as: symptoms, admittance to hospital, use of (additional) medication, adjustments of or the need for (non)-invasive ventilatory support and other medical interventions. In addition, a standardized questionnaire about SARS-CoV-2 vaccination and experienced side effects was sent to patients who were eligible for vaccination on 16–7-2021.-2021 (age ≥ 17 years). Consent on reporting the disease course was obtained from each patient.

## Results

### Patient characteristics (Table 1)

**Table 1:** Overview of patients with Pompe disease with a proven SARS-CoV-2 infection

Patient; Pompe type	Age	Gender	Age at diagnosis	Age at start ERT	Pompe disease severity	Recent immune modulation	BMI	FVC sitting/supine before infection	FVC reduction after infection	COVID disease duration (days)	Symptoms	Long-term symtoms	Chest X-ray	SARS-CoV-2 antibody titer
1. Classic infantile	8	F	4 months	4 months	Moderate. Responsive to ERT	No	20.7	67/no recent measurement	No	7	Cough, headache	No	n/a	n/a
2. Classic infantile	8	F	8 days	10 days	Moderate. Responsive to ERT	No	17.6	91/74	n/a	3	Fever, headache, ear pain	No	n/a	n/a
3. Classic infantile	11	F	3 days	8 days	Moderate. Responsive to ERT	No	20.3	86/87	n/a	2	Tiredness, sore throat	No	n/a	n/a
4. Classic infantile	16	F	6 days	16 days	Moderate. Wheelchair bound	No	22.3	56/no recent measurement	Yes; normalized after 1 month	7	Cough, stuffed nose, left-sided muscle weakness	Left-sided muscle weakness	n/a	n/a
5. Childhood	20	M	2 years; 4 months	9 years; 9 months	Mild	No	19.9	94/90	No	15	Sore throat, stuffed nose, general weakness, anosmia, loss of taste	No	n/a	n/a
6. Classic infantile	22	F	12 years; 3 months	No ERT	Mild	No	21.5	90/86	n/a	8	Fever, cough, anosmia, headache, stuffed nose	No	n/a	n/a
7. Classic infantile	22	M	20 days	4 months	Severe. Non-invasive ventilation, wheelchair bound	No	24.6	27/not possible	No	n/a	No symptoms	No	n/a	n/a
8. Childhood	22	M	3 years; 6 months	6 years	Mild	No	25.1	79/68	No	6	Cough, anosmia	Anosmia	n/a	n/a
9. Childhood^a^	29	F	15 years	23 years	Mild	No	30	125/115	No	10	Cough, stuffed nose, myalgia, headache, fatigue	Fatigue	Normal after recovery	n/a
10. Childhood	29	M	2 years	15 years	Mild	No	23	102/94	No	16	Cough, stuffed nose, anosmia, loss of taste	Anosmia	n/a	Positive 1,7 months after first symptoms
11. Late onset	40	F	24 years	26 years	Moderate. Use of nightly non-invasive ventilation	No	26	50/25	No	14	Fever, dyspnea, fatigue, myalgia, nausea, chest pain, anosmia, loss of taste	Fatigue, anosmia, chest pains, pain between shoulders, palpitations	n/a	Positive 4 months after first symptoms
12. Late onset	49	F	37 years	38 years	Mild	No	27	61/34	No	19	Cough, myalgia, fever, chest pain, sore throat, headache, diarrhoea	Fever, dyspnea during exercise, heart palpitations	Normal after recovery	n/a
13. Late onset	49	F	36 years	38 years	Moderate. Use of walking aid	No	27.9	82/50	Yes, asymptomatic, no new spirometry yet	7	Myalgia, nausea, bloated feeling	No	n/a	n/a
14. Late onset	60	M	56 years	Discontinued in 2018	Mild	No	27	83/78	No	9	Cough, fever, headache	No	n/a	n/a
15. Late onset^a^	62	F	59 years	59 years	Mild	No	28.1	82/64	No	n/a	No symptoms	No	n/a	n/a

^a^Two patients were infected after receiving their first dose of vaccination. Mild disease severity: classified as no use of ventilator or walking aid

*N*/*a* not available

At the time of writing, November 2021, *fifteen* patients with Pompe disease have had a SARS-CoV-2 infection (8.9% of our cohort). Only two patients were tested positive after the first dose of vaccination, one a day after vaccination (due to symptoms and contract tracing) and one after 9 days (due to contact tracing). To date no infections have been reported after patients were fully vaccinated. These fifteen patients include *five* patients with classic infantile form (age 8–22) and *ten* with LOPD, both childhood or adult (age 20–62). Pompe disease duration varied from 8 to 23 years. All but two patients were treated with ERT. Table 1 shows the patient characteristics, age at Pompe diagnosis and start ERT, age at infection, SARS-CoV-2 infections, lung function tests and radiologic examinations before and after SARS-CoV-2 infection if obtained during the pandemic are presented in Table 1.

*Ten* of the 15 patients were mildly affected with Pompe disease, meaning none of these patients used either ventilators or walking aids.

*Five* patients showed moderate to severe symptoms, meaning they were either a classic infantile patient responsive to ERT or a patient using a ventilator or walking aid.

### Risk factors

Body mass index ranged from 17.6 to 30 kg/m^2^. One patient with classic infantile and six late onset patients were overweight (BMI ≥ 25). Two patients with classic infantile Pompe disease had been treated with immune modulation several years prior to the infection, but had a fully functioning immune system at the time of infection. There were no other relevant comorbidities or risk factors. Thirteen patients had a reduced pulmonary function prior to infection.

### SARS-CoV-2 disease symptoms and duration

*Fourteen* patients COVID-19 were diagnosed with a positive RNA-PCR test. One patient was diagnosed in retrospect by testing for SARS-CoV-2 antibodies four months after initial symptoms.

One severely affected classic infantile patient (aged 22) and one late onset patient (aged 62, infected after first dose of vaccination and tested as a result of contact tracing), reported no symptoms. No patients were admitted to hospital due to infection. No patients were prescribed drugs to treat the infection. Three patients reported worsening of their dyspnea while physically active (e.g. walking or climbing stairs). Reported symptoms were coughing, anosmia and diminished taste, fever, a runny or stuffed nose, sore throat, headache, myalgia, chest pain, nausea, and diarrhea. The SARS-CoV-2 disease duration, calculated from the first day of symptoms to the first day of feeling well according to the patient, varied from 2 to 19 days after which normal daily life was resumed. Longer lasting symptoms experienced while already participating in daily life activities were anosmia, fatigue, aspecific chest and back pains, myalgia, palpitations and mild shortness of breath during physical activity. These symptoms lasted a maximum of four months. Six patients reported no longer lasting symptoms. (Table 1).

During infection, two infusions with ERT were canceled and two were postponed.

### Pulmonary function before and after infection

Two patients showed a reduced Forced Vital Capacity (FVC) after infection without symptoms, compared to before infection (7–12% lower), which was normalized after 1 month in the patient with classic infantile Pompe disease. These decreases may also be explained by normal variation and/or decline caused by Pompe disease itself. The change in FVC did not lead to the need of (additional) ventilation.

### Other

One classic infantile form female patient (aged 16) who was wheelchair dependent, with preexistent asymmetry of muscle weakness, more pronounced at the left side, experienced subacute progression of weakness of the left arm and leg during infection. Based on clinical course and MRI of the brain, no thrombo-embolic neurological complications were established and these symptoms were attributed to temporarily worsening of pre-existent weakness due to the SARS-CoV-2 infection. These symptoms eventually subsided after six months.

### Vaccination against SARS-CoV-2

A total of 122 patients > 17 years of age and eligible for vaccination on 16-7-2021 received a questionnaire regarding vaccination. 51 patients returned the vaccination questionnaire (41.8%) of which 50 indicated that they had received a vaccination (98%). Forty-eight of these patients received two doses of either Moderna (n = 37), Pfizer (n = 8), or AstraZeneca(n = 3). One patient received only one dose because he had experienced a SARS-CoV-2 infection (according to Dutch guidelines for COVID vaccination at the time [14]). One questionnaire was sent back without patient study number and did not provide information on the type of vaccination used. 60% of patients mentioned side effects related to the vaccination, such as: pain at injection site (n = 16), headache (n = 12), myalgia (n = 11), fever (n = 9), fatigue (n = 8) and swollen lymph nodes (n = 3). Of note one patient reported need for increased need for ventilation during the day of vaccination, which subsided after a couple of hours. One patient developed perimyocarditis two weeks after the first Moderna dose. As no other causes for the perimyocarditis were found and perimycarditis is now a known rare side effect of mRNA COVID vaccination (and of SARS-CoV-2 infection itself) this was probably caused by the vaccination [15]. She recovered and received her second dose as scheduled.

## Discussion

We present a first clinical insight into the course of SARS-CoV-2 infection in Pompe patients. Even though patients are regarded to be at medium to high risk for severe COVID-19 [11], patients generally showed mild symptoms of the infection. No patients needed to be admitted to a hospital.

Moreover, in contrast to what we expected, no patients showed a lasting decline in muscle function due to the disease or concomitant suboptimal intake of nutrients. Only one patient experienced temporary worsening of left sided muscle weakness without an acute neurological substrate. One patient developed a perimyocarditis two weeks after her first dose of vaccination, which has been reported as a rare side effect of the Moderna vaccination [16, 17]. All other reported side effect were mild.

However, only 9% of our Pompe population has been infected with SARS-CoV-2, and the infected patients were relatively young and mostly mildly affected by Pompe disease. No patients with an impaired immune system were infected (“Appendix”).

As about 30% of the Dutch population already had infection related antibodies in June 2021, the 9% confirmed SARS-CoV-2 infection rate in our Pompe cohort in November 2021 is lower than expected [18]. This is likely caused by a stronger awareness of self-protection in Pompe patients than in the normal population, because patients know they are considered to be at risk of developing severe SARS-CoV-19. This is certainly what we observed clinically in the patients more severely affected by Pompe disease in our cohort. Therefore, even though we observed a mild course of SARS-CoV-19 infection in our patients, we do not know whether patients severely affected by Pompe disease have an increased risk for severe COVID-19. Until this is clarified, we recommend to regard Pompe disease as a risk factor for severe COVID-19 and treat patients accordingly, and strongly recommend vaccination for all patients, as vaccination has been proven to be safe and effective in preventing severe COVID-19.

## Data Availability

Data sharing is not applicable to this article as no datasets were generated or analysed during the current study.

## Appendix

See Table 2.

**Table 2:** Overview of patients with Pompe disease and relevant risk factors for SARS-CoV-2 disease in the Erasmus Medical Center

Pompe type	No. of patients	No. of patients overweight	No. of patients with ventilator support	No. of patients with compromised immune system during the pandemic
Classic infantile	21	7	5	3
Late onset; childhood presentation	23	8	0	0
Late onset; adult presentation	125	69	49	0
Total	169	84	54	3

## Abbreviations

CLMD: Center for lysosomal and metabolic diseases
COVID-19: Coronavirus disease 2019
ERT: Enzyme replacement therapy
FVC: Forced vital capacity
GSDII: Glycogen storage disease type II
LOPD: Late onset Pompe disease
SARS-CoV-2: Severe acute respiratory syndrome coronavirus 2
